# The role of the Internet in later life autonomy: Silver surfers in Spain

**DOI:** 10.1057/s41599-023-01536-x

**Published:** 2023-02-13

**Authors:** Carmen Llorente-Barroso, María Sánchez-Valle, Mónica Viñarás-Abad

**Affiliations:** 1 Department of Applied Communication Studies, Complutense University of Madrid, Madrid, Spain; 2 Department of Audio-visual Communication and Advertising, CEU San Pablo University, Madrid, Spain

**Keywords:** Cultural and media studies, Social policy

## Abstract

The new digital panorama has enhanced the importance of the Internet, as well as Information and Communications Technology (ICT), in developing a society in which seniors play a proactive role. The main purpose of this article is to define a taxonomy of silver surfers according to the ways they use the Internet and ICT, with a special focus on e-commerce and e-government. A quantitative methodology was used, based on the study of 405 Spanish internet users between 60 and 79 years of age, which was conducted by telephone in February of 2019. Seven groups were identified through a combination of dimensionality reduction techniques and cluster analysis. The results indicate neither a consistent pattern in the specific ways older adults use the Internet nor a homogeneous level of digital knowledge among this demographic group. To some extent, this is a result of disparities in both the perception of digital security that seniors associate with e-commerce and/or e-government and the level of trust engendered by such operations. The Able and Daring are the most numerous clusters, which coincide with the categories of the most active and prepared users. The Sceptical take third place in terms of number of users, as they display limited use of the Internet and claim to have a low digital skills level. However, carrying out both online shopping and administrative procedures without the need for assistance is becoming increasingly frequent among all of those surveyed.

## Introduction

Nowadays, the ageing of societies is one of the most pressing challenges for developed countries. The concern to maintain a state of well-being has led to deep reflection directed at maximising autonomy throughout the last stages of life. In the 1980s, the European Union initiated the design and implementation of a new policy on ageing that fostered proactivity among seniors in order to provide them with enhanced well-being and contribute to the sustainability of European social security systems (Walker, 2009).

Data available prior to the COVID-19 pandemic indicated that in Europe and North America, the percentage of people aged 65 and over would increase from 18% in 2019 to 29.3% by 2100 (United Nations, 2019). Despite the devastating effects of the pandemic, at the beginning of 2022 this age group reached 20.08% of the total Spanish population, according to the latest results published by the National Institute of Statistics (INE) in Spain, which registered the percentage of the population over 80 years of age at 6.08% (INE, 2022). Furthermore, the demographic change in birth and death rates has led to the creation of age-friendly health care systems focused on preventive action in order to achieve healthy, sustainable ageing (Naja et al. 2017). Another effect has been the establishment of systems in which factors of social participation and quality of life incentivise greater efficiency in senior care services (Lindsey-Brett et al. 2019).

At the same time, connecting to the Internet continues to be a rising trend in Spanish society, with an access rate at 91.3% of the entire population over the past three months (ONTSI, 2021). In general, although the probability of having access to the Internet declines with old age (Huxhold et al. 2020; Olsson et al. 2019), the generational digital divide is progressively being reduced by increased contact with ICT by the new generation of seniors (Gilleard and Higgs, 2008; Lüders and Gjevjon, 2017). Nevertheless, digital exclusion continues to affect the older population. According to the latest data from the INE (2021), only 56.3% of Spanish adults between 65 and 74 years of age, 25.9% between 75 and 84, and 8.7% over 85 years of age have used the Internet on a daily basis in the past year. Moreover, significant differences in senior internet use continue to be observed, with those over 65 performing only basic tasks (INE, 2021). Given this panorama of ageing, the Internet and ICT have emerged as instruments of empowerment and inclusion for seniors in society (Sánchez-Valle et al. 2017). However, societal digitisation is leading to a situation in which the participation of citizens is determined by their technological skill. As a result, those without such abilities, which is the case with some seniors, are socially excluded (Holgersson et al. 2019). The consequence is a reduction of opportunities for relationships and a weakening of social, economic, political, and cultural rights (Burholt et al. 2020; Mihelj et al. 2019).

Previous research shows not only the negative impact of age on people’s level of digital knowledge and use of ICT (Hargittai, 2002; Helsper and Van-Deursen, 2015; Hunsaker and Hargittai, 2018; Olsson et al. 2019; Ragnedda et al. 2020; Van-Deursen and Helsper, 2018; Van-Deursen and Van-Dijk, 2019). It also points to the existence of intra-generational inequalities that define different profiles of older internet users, confirming the heterogeneity of this social group at the technological level (Elueze and Quan-Haase, 2018; Gallistl and Nimrod, 2020; Hänninen et al. 2021; Näsi et al. 2012; Mason and Pereira, 2011; Nimrod, 2017; Quan-Haase et al. 2018; Van-Deursen and Van-Dijk, 2011; Vulpe and Crăciun, 2020). In recent years, relevant scientific contributions have been made, ranging from binary classifications between senior internet users and non-users (Van-Deursen and Helsper, 2015a), to more complex and specific typologies that differentiate various clusters of silver surfers. Such differentiation has been determined according to their digital knowledge and the uses they make of online media (Quan-Haase et al. 2018; Vulpe and Crăciun, 2020), as well as their online search behaviour (Mason and Pereira, 2011), media consumption (off and online), leisure preferences (Nimrod, 2017), concerns about online privacy (Elueze and Quan-Haase, 2018), and an assortment of media-based leisure activities (Gallistl and Nimrod, 2020). However, these studies do not consider certain variables that may be decisive in defining some profiles of digital seniors, nor do they take into account certain specific uses of ICT, such as e-government and e-commerce, which are vital in developed societies and can offer valuable indirect benefits to users. This paper aims to provide greater understanding of a social group that is highly complex and diverse in its digital use. It follows in the footsteps of previous research that has focused on the digital skills of older adults and their use of digital media (Quan-Haase et al. 2018; Vulpe and Crăciun, 2020), yet it incorporates trust and security as discriminating factors as well. In addition to the most common uses of the Internet, in order to develop a typology of silver surfers, specific uses that traditionally have not been widespread among this population have been considered as well. These have been scarcely explored, yet they are increasingly important in the life of all citizens. Moreover, such uses determine their digital inclusion or exclusion in developed societies. In this regard, the use of e-commerce, e-government, online banking, and social networks by older adults has been examined, with a special focus on WhatsApp, due to its widespread acceptance among this demographic group (Fernández-Ardèvol and Rosales, 2018; Rosales and Fernández-Ardèvol, 2016). The classification offered in this article reflects the high level of complexity of an exceedingly heterogeneous social group, differentiating up to seven clusters of internet users over the age of 60. These individuals have diverse levels of technological skill, show varying degrees of trust toward the Internet, and they use the Web in various ways.

Consequently, the main purpose of this study is to create a taxonomy of seniors as internet users (silver surfers) according to the tasks they perform through this channel, with special emphasis on their use of e-commerce and e-government, in addition to their level of trust in using the Web. To achieve the overall objective, three specific objectives (SO) have been established as follows:

SO1. Identify the differences between the diverse types of seniors as internet users according to the ways they use the Web and their level of skill and trust in using it.

SO2. Discover and define the profiles of internet users within this age group.

SO3. Find the attributes of users over 60 years of age who feel more insecure on the Internet and admit to having a lower level of digital knowledge, as they are the most vulnerable group, require more attention, and need to increase their level of knowledge in this area.

## Literature review

### The digital divide and heterogeneity among older adults in the online environment

Over the last two decades, research on the digital divide has redirected its focus by acknowledging three levels based on the focal point of analysis. In the 1990s, a debate on the so-called first-level digital divide began, which identified people with and without the physical, material, or technical access to the Internet and ICT, differentiating between users and non-users (Hargittai, 2002; Hargittai, 2003; Van-Deursen and Van-Dijk, 2015). When the Internet became available to a large percentage of the population, this dual classification became simplistic, and divergences in the digital skills level and uses of the Internet were observed, which has resulted in inequalities in the ability to use the Internet efficiently and has provided evidence of a second-level digital divide (Hargittai, 2002; Hargittai, 2003; Van-Deursen and Van-Dijk, 2014; Van-Deursen and Van-Dijk, 2015). The level of online skill depends on factors beyond mere connectivity, such as the quality of technical means, user autonomy, the social support they receive, and their digital experience (Hargittai, 2003). Moreover, Van-Deursen and Van-Dijk (2009, 2011) have identified four categories of internet skills as follows:

1. Operational internet skills, associated with basic skills in using the Internet.
2. Formal internet skills, linked to navigation in the hypermedia structure of the Internet
3. Informational internet skills, related to satisfying the information needs of the user.
4. Strategic internet skills, related to the ability to use the Internet as a tool to achieve goals that improve one’s position in society.

More than a decade after the first research on the second-level digital divide, Van-Deursen and Helsper (2015b) proposed an operational framework for measuring the benefits that the Internet provides to users in multiple areas of life (economic, social, cultural, and personal). At the same time, these authors considered the inequality resulting from a third-level digital divide, which shows that people with higher social status make more productive use of the digital environment. Differences in material access to the Internet (first-level digital divide) imply differences both in the level of digital skills and in the type of uses made of the Internet (second-level digital divide), and in the degree of inequality in the attainment of benefits derived from those uses (third-level digital divide). Thus, using a wider variety of devices is associated with performing a larger assortment of online tasks and obtaining better results (Van-Deursen and Van-Dijk, 2019).

Age is a factor that tends to negatively impact the digital divide of the three levels. Older adults tend to own a smaller variety of devices (first-level digital divide) (Van-Deursen and Van-Dijk, 2019), reflect a lower level of digital skills and engagement with the Internet (second-level digital divide) (Hargittai, 2002), and tend to achieve fewer tangible results from digital media use in all areas of their lives (third-level digital divide). As such, younger users benefit more from digital media use (Van-Deursen and Helsper, 2015, 2018).

At the beginning of the current century, the limited use of technology by this segment of the population was associated with the minimal importance they placed on it, as well as the limited advantages they perceived in its use (Selwyn et al. 2003). The stereotypical image of older people as less tech-savvy and not as interested in technology has reinforced the “grey digital divide” (Ivan and Cutler, 2021, p.134). With regard to technology, ICT access and literacy decrease with age (Hunsaker and Hargittai, 2018; Olsson et al. 2019). In fact, digital knowledge “is negatively related to age” (Ragnedda et al. 2020, p.804), a correlation resulting in a persistent digital divide (Neves et al. 2018).

Along these lines, Nimrod (2017) detected a second-level digital divide among seniors, finding that only a minority of silver surfers (17.7%), or those labelled as Heavy Asynchronous Media Users, intensely engage in new media practices involving social media. Moreover, Van-Deursen and Van-Dijk (2011) have observed that age has a strong impact on the decline of operational and formal internet skills, yet this is not a significant factor in skill level with regard to obtaining information or using the Internet strategically. Nevertheless, previous studies show that age is not the most determining factor in internet use, pointing out that performing online tasks daily may have a greater impact on digital behaviour (Hill et al. 2011), and on the browsing patterns of older web users (Ivan et al. 2020; Loos, 2011; Loos and Ivan, 2022). Loos (2012) argues that age is not the only factor that plays an important role in the use of technology by older people. Other relevant factors include the various stages of life, ways of socialising during the ageing process, and age-related functional limitations. In the past few years, many seniors have found online activity to be more interesting than other daily routines, with exceptional value being placed on using the Internet to communicate with their families, an aspect that could be used to entice reticent seniors to start using the Web (Jones et al. 2015).

Moreover, socio-economic status and access to technology in the home fosters use and enhances the opportunity to use the Internet regularly in old age (Sylvia and Szydlik, 2005). As noted above, experience using technology generally has a correlation with a person’s level of digital skill, thus helping to reduce the second-level digital divide (Hargittai, 2002). Indeed, digital experience is a more important factor than age in senior internet use (Loos, 2012). An experimental study by Hill et al. (2011) shows the impact of computer experience on the digital behaviour of older adults. Along the same lines, Loos (2011, p.200) observes that “the frequency of internet use impacts more heavily on our navigation patterns than does age”. Typically, seniors who have had previous contact with technology are more motivated to learn about ICT. Moreover, they view it as an extremely useful tool, as it offers this segment of the population independence, confidence, and social commitment (Baker et al. 2017).

Previous scientific literature regarding the impact of the digital divide on older adults has focused on diverse factors in order to determine the existence of inequalities in terms of digital use and skills. However, the development of new uses and the progressive digital dependency require further research, as well as an answer to the following question, which is related to SO1:

RQ1. How do diverse types of older internet users differ in terms of their internet use, level of skill, and online trust?

### Typologies of older internet users

In European society, clear differences remain in the active use of the Internet by the senior population, which highlights a lack of homogeneity in the use that seniors make of the Web (Näsi et al. 2012). According to Hänninen et al. (2021, p.13), “The heterogeneity of ICT use among older adults manifests itself more as a practical response to the complexity of the constantly changing ICT landscape” than to their limited digital skills or lack of interest in technological advances. This heterogeneity among older internet users and non-users suggests that within the context of the digital divide, there are gradations or variations of digital inclusion rather than gaps (Van-Deursen and Helsper, 2015a, Van-Deursen and Van-Dijk, 2015). This situation has led to various classifications of silver surfers, or digital seniors, according to different criteria.

Van-Deursen and Helsper (2015a) have examined the differences between adults over 65 who are internet users and those who are not. They found that even when older non-internet users live in environments with access to the Internet, many of them are unwilling to use it, either due to a lack of interest or a lack of digital knowledge. Moreover, they exhibit significant differences related to gender, age, education, household structure, and attitude toward the Internet. The results of these authors suggest that the use of this medium is dominated by younger men within the senior group, which reveals that attitude toward the Internet is a determining factor in the way older people use it (Van-Deursen and Helsper, 2015a).

Beyond dichotomous classifications, Mason and Pereira (2011) show the heterogeneity of internet users over the age of 60 according to the skills and attitudes they display in their online information-seeking behaviour, differentiating among the following:

1. Rejecters: They deliberately use technology in a limited way because of their lack of trust in the Internet, their disdain for technology, and the perceived poor investment of their valuable time.
2. Utilizers: These users develop practical ways to use the Internet in order to save time or money when it comes to safe tasks (e-mail, search engines, online banking, and e-commerce).
3. Browsers: These individuals manifest frequent, diverse, and adventurous use of the Internet as a source of information, entertainment and social contact.
4. Augmenters: They use the Internet in a way that is similar to that of digital natives, as they consider the Internet to be an integral part of their lives with the intention of creating their own digital footprint.

For her part, Nimrod (2017) has studied the degree to which adults over 60 have changed their consumption of traditional media for more innovative communication practices, applying a cluster analysis that allowed her to differentiate four sub-segments of silver surfers according to their media habits (time spent using the media and each communicative practice), socio-cultural background, and leisure preferences:

1. Heavy Synchronous One-to-Many Users (26.6%): They show heavy use of one-to-many forms of communication (387 min per day on average) and relatively low use of asynchronous one-to-many ways of communicating.
2. Heavy One-to-Many Users (11.8%): They display intense use of both synchronous and asynchronous forms of one-to-many communication, with a very high overall level of time spent online (972 min per day on average).
3. Heavy Asynchronous Media Users (17.7%): They make heavy use of asynchronous communication associated with the new media.
4. Light Users (43.9%): These individuals make relatively light use of communication media and have a preference for non-media forms of social activity.

Quan-Haase et al. (2018) propose a taxonomy of internet users aged 65 and over based on 41 in-depth interviews, according to their use of digital media and their level of technological skill, as follows:

1. Non-users (10%): They do not have digital skills nor perform online tasks.
2. Reluctant Users (17%): They show a low level of digital skill, which allows them to perform between zero and two basic online activities (e-mail, paying bills, or carrying out searches). Moreover, they have a low level of confidence in trying new activities, and they prefer the phone for interacting with loved ones.
3. Apprehensive Users (17%): These individuals claim to have low-level digital skills, although they perform between three and six online activities as part of their usual routine (e-mail, Skype, and searches).
4. Basic Users (27%): Acknowledge an average level of digital skills for performing one or two online activities, and although they feel more comfortable than Apprehensive Users on the Internet, their use of digital media is more restricted.
5. Go-Getters (22%): They have an average level of digital skill, yet they carry out between three and six online activities; their curiosity about the Internet motivates them to experiment with various uses (e-mail, Skype, Facebook, and searches).
6. Savvy Users (7%): They have a high level of digital skills and participate frequently in a wide range of online tasks (more than three, including online banking and the use of Facebook). This is the senior profile in this classification that spends the most time on digital media.

Elueze and Quan-Haase (2018) distinguish five categories of older internet users according to their attitudes and concerns about privacy in the digital space, based on 40 interviews with adults aged 65 and over:

1. Fundamentalists (13%): These people are very possessive of their privacy to the point of paranoia about the risks associated with disclosing personal information over the Internet, and they have a high level of anxiety and fear of being hacked or scammed.
2. Intense Pragmatists (15%): They are worried about their privacy in some respects, although they understand that there must be a balance between privacy protection and the use of digital media (e-commerce). Nevertheless, they are cautious about carrying out certain tasks (e-banking).
3. Relaxed Pragmatists (42%): They define themselves as protective of their privacy in certain aspects. They have a basic understanding of the risks involved in sharing data online, and they express their concerns, yet it does not prevent some of them from making online purchases. However, others in this group hold back in their general use of digital media.
4. Marginally Concerned (25%): These users are not possessive of their privacy because they feel that their digital activity does not make them vulnerable. They understand the balance between participating on the Internet and protecting their privacy when shopping online. They are not very concerned about scams and spam, although they consider themselves cautious about disclosing personal data.
5. Cynical Experts (5%): They are distrustful of certain aspects of their privacy in the online environment, although they disseminate information about themselves online. Their scepticism leads them to be concerned about spam, government surveillance, and access to unauthorised data by potential fraudsters when carrying out internet banking.

Vulpe and Crăciun (2020) propose a typology of people over 65 according to their use of ICT and their specific needs in the digital environment, identifying three groups as follows:

1. Digitally Immersed Communicators: These users are fully integrated in the digital environment, making diverse and frequent use of ICT, including telephony that is landline-based, mobile, and online, in addition to instant messaging and social networks, for the purpose of communicating and passing the time.
2. Asynchronous Communicators: They are aware of the importance of ICT, although they use it less frequently than Digitally Immersed Communicators, and they prefer more traditional means of communication (landlines, mobile phones, and e-mail).
3. Phone Enjoyers: They accept the use of ICT, but their interest in technology is almost exclusively related to the telephone (landline and mobile). As such, they do not take advantage of digital services and are reluctant to participate in social media.

Gallistl and Nimrod (2020) developed a taxonomy of internet users over 60 years of age based on a cluster analysis that identifies four groups of senior internet users according to their characteristics, preferences, and assortment of media-based leisure activities:

1. Innovative Traditionalists (8.8%): These users consume traditional content through digital formats on a frequent basis (online news, online radio, and digital books), spending an average of 10 hours per day on media-based leisure activities.
2. Entertainment Seekers (16.1%): They participate in social networks and are heavily involved in media-based leisure activities (online and offline), spending an average of 11.9 hours per day on such pursuits.
3. Selective Content Consumers (26.1%): They participate intensely in media-based leisure activities (9.7 hours per day on average), but their selection is related to the type of content consumed, which is mostly online news and video on demand.
4. Eclectic Media Users (49%): These users do not display a specific pattern of participation in media-based leisure activities, nor do they spend a lot of time each day on such endeavours (6.8 hours on average).

These earlier studies have made valuable contributions to the knowledge of silver surfers. Nevertheless, the constant development of technology and the emergence of digital uses that are becoming increasingly essential, yet less widespread among seniors (e-commerce, e-government, and social media), due to a lack of trust in the Internet, require these findings to be enhanced. In this regard, the next question arises in relation to SO2:

RQ2. Is it possible to suggest a classification of silver surfers that offers new categories, when taking into account the new digital needs and demands?

### The potential value of technology and digital knowledge for active ageing

Active ageing has been defined by the World Health Organization (WHO, 2002, p.2) as “the process of optimizing opportunities for health, participation, and security in order to enhance the quality of life as people age”. In its psychological aspect, this concept is linked to successful ageing (Rowe and Khan, 1997), which identifies participatory social functioning as one of its domains, and is related to the experience of old age from the perspective of an active commitment to life. One’s own perception of age also plays a role. Bernhold (2021) reveals that seniors behave differently according to their perception of age, including their relationship with the media. Successful ageing depends on individuals’ adoption of healthy lifestyles in which they are socially integrated. Moreover, families and communities can contribute by providing support in order for seniors to implement these habits into their lives (Subramaniam et al. 2019). The concern for active ageing persists in a world with an increasingly older population. Thus, both the WHO (2020) and the European Agency for Safety and Health at Word (2021) have recently declared the ten-year period from 2020 to 2030 to be the Decade of Healthy Ageing, with a commitment to improving the quality of life of older people in order to achieve a sustainable world.

Furthermore, it has been found that regardless of people’s socio-economic or socio-cultural attributes, their level of digital skills and the activities they carry out online can provide them with indirect benefits that are economic, cultural, social, and personal (Van-Deursen and Helsper, 2018). However, online activities that have important benefits for older people’s offline lives, such as the use of public services, require technological expertise that is more complex than what is needed for simple, everyday activities such as searching for information (Van-Deursen and Helsper, 2015a). In general, personal and social uses of the Internet are the ones that bring the most indirect benefits to users. Nevertheless, operational internet skills, which are often the focus of social policy and intervention, are not directly related to the achievement of outcomes from internet use. Therefore, they do not offer major breakthroughs in ending the digital inequalities linked to the achievement of various benefits (third-level digital divide) (Van-Deursen and Helsper, 2018).

From a sociocultural perspective, active internet use by seniors is correlated with diverse leisure activities for this age group; seniors who engage in a variety of leisure activities are more likely to be internet users than those who participate in less activities throughout the day (Näsi et al. 2012). At the personal level, connecting to the Web enhances the health and mental well-being of seniors, and it gives them a greater sense of belonging to the community, thereby alleviating loneliness through the opportunities provided by the Internet (Jones et al. 2015). Seniors over 65 associate the feeling of old age with loneliness, due to a lack of projects, especially among women, and to boredom, especially among men (Bordone et al. 2019). This is particularly true of seniors who are suffering from an illness that limits their mobility. As such, ICT facilitates social, cultural, and economic participation even more (Malanowski et al. 2008). In addition, Iván and Fernández-Ardèvol (Ivan and Fernández-Ardèvol, 2017) point out that the opportunities offered by ICT in allowing older people to communicate with their children and grandchildren when physically separated from them encourages seniors to learn more about the use of these digital applications. In this sense, ICT facilitates intergenerational connection (Freeman et al. 2020; Llorente-Barroso et al. 2021), which allows families to feel a sense of togetherness despite being physically apart (Taipale, 2019). For older people, however, this type of digital connection does not achieve the level of emotional satisfaction produced by in-person contact with family and friends (Llorente-Barroso et al. 2021). Moreover, older people’s interest in learning to use internet-based communication decreases when families are once again able to be together in-person (Ivan and Fernández-Ardèvol, 2017).

In general, numerous studies have pointed out the opportunities offered by the Internet and ICT in promoting active, autonomous, and inclusive ageing (González-Oñate et al. 2015; Llorente-Barroso et al. 2015; Shapira et al. 2007; Tirado-Morueta et al. 2016). Such opportunities empower seniors and enable them to participate in society (Sánchez-Valle et al. 2017), thereby allowing them to achieve tangible results in different areas of their lives (Van-Deursen and Helsper, 2018).

### Education, social-family support, and e-inclusion as responses to the generational digital divide

The evolution of scientific research on digital inclusion has gone hand in hand with the study of the digital divide. The debate on inclusion initially focused on access (infrastructure); next, it placed emphasis on skills, motivation, and commitment of use; and finally, it shifted toward tangible (offline) results associated with digital media use (Helsper and Van-Deursen, 2015). Unlike age, the variables of education and attitude toward the Internet are the ones that influence digital knowledge and help reduce inequalities driven by the digital divides at all three levels. Attitude toward the Internet plays a key role both in material access (first-level digital divide) and the development of digital skills and uses of the Internet (second-level digital divide). Moreover, attitude is even more decisive than many socio-economic variables, especially when starting to use the Internet (Van Dijk, 2005). Education is also related to one’s level of digital capability (Hargittai, 2002), contributing significantly to the development of all categories of internet skills (operational, formal, informational, and strategic) (Van-Deursen and Van-Dijk, 2011). Furthermore, education plays a key role in the third-level digital divide, as people with a high level of education take greater advantage of the Internet than those with less education (Van-Deursen and Van-Dijk, 2014), especially in tasks related to e-commerce (Viñarás-Abad et al. 2022), e-government (Sánchez-Valle et al. 2022), and online education (Van-Deursen and Helsper, 2015). Overall, this indicates a relationship between education and the achievement of economic and personal goals (Van-Deursen and Helsper, 2018).

Moreover, these barriers need to be addressed in order for this demographic group to participate in the online society (Baker et al. 2017). One of the key factors in maximising the benefits of ICT is education; for this reason, it is essential to train seniors in specific digital skills (Abad-Alcalá, 2014). Furthermore, participation in digital education programmes is associated with performing specific tasks, such as those related to e-government, especially in the case of vulnerable citizens such as seniors and the disabled (Lee and Porumbescu, 2019; Kolotouchkina et al. 2022; Sánchez-Valle et al. 2022). Ongoing education in technical competence for seniors would improve their quality of life (Tirado-Morueta et al. 2016), yet it requires the involvement of young people and institutions (Barrantes-Cáceres and Cozzubo-Chaparro, 2019). Moreover, in order to overcome the digital exclusion of seniors, socio-educational measures should consider psychological and social barriers as well as individual motivations and socio-cultural preferences, due to the fact that technically speaking, many older non-internet users live in places that allow internet access, but they are unwilling to use it (Van-Deursen and Helsper, 2015a; Van-Deursen and Van-Dijk, 2014).

In all probability, due to the generational differences noted above, older adults are more likely to seek social support for digital media use than younger people, yet age negatively impacts both the range of informal support available and the quality of that support. This means that older people are less likely to have a diversity of effective support, and they will continue to need help when the same types of problems reoccur, thereby hindering them from achieving self-sufficiency (Helsper and Van-Deursen, 2017).

The setting in which seniors live also affects their internet use, as older adults who live alone and are not internet literate are less likely to start using the Internet (Van-Deursen and Helsper, 2015a). Moreover, it has been shown that seniors are strongly influenced to use technology by people close to them, whether family members or friends (Friemel, 2016). This is especially true of certain online tasks such as e-government (Chirara, 2018; Yap et al. 2017; Sánchez-Valle et al. 2022), which in the case of seniors enables the development of autonomy and quality of life (Siren and Knudsen, 2017).

The last two sections of the literature review point out the value of ICT and digital knowledge in providing indirect benefits to different areas of older people’s lives. However, inequalities arising from the second-level digital divide seem to have an impact on the third-level digital divide, which prevents some older adults from benefiting from the tangible results of competent internet use. In relation to SO3, this research aims to answer the following question:

RQ3. What characteristics define the most insecure and least empowered users over 60, and how can these aspects be counteracted so that seniors might benefit from the different opportunities offered by the Internet, thereby contributing to successful ageing?

## Material and methods

### Sample and data collection procedure

This research is part of two competitive projects with public funding that address the situation of digital vulnerability of older adults in Spain. This is a key issue in light of the proliferation of ICT and the emergence of new scenarios that require internet use in many areas of life in developed societies, which are characterised by continuous online connection. Specifically, to achieve the objectives set out in this article, a quantitative methodology was designed and developed based on a survey of Spanish internet users aged 60 and over. This age was selected according to the official United Nations (2013) definition of older people (European Union, 2019), which has been used in previous studies to describe different profiles of silver surfers (Gallistl and Nimrod, 2020; Mason and Pereira, 2011; Nimrod, 2017).

The survey was carried out using a random finite sample of 405 Spanish men and women over the age of 60 who are internet users. In defining the sample, quotas were established by gender, age, and place of residence in order for the sample to be representative of the Spanish population aged 60 and over who use the Internet. Thus, the characteristics of the sample provided a confidence level of 95% with a sampling error of PQ = 0.50 of 4.9% (PQ = 0.75 → 4.2%, and PQ = 0.90 → 2.9%), which guarantees the reliability of the results for their extrapolation to the population under study (Spanish internet users aged 60 and over). The distribution of participants in terms of gender is 58.8% men and 41.2% women. By age, the distribution is as follows: from 60–64, 30.9%; from 65–69, 24.7%; from 70–74, 36.3%; and from 75–79, 8.1%. Regarding the composition of the sample according to the place of residence of the participants, adjustments had to be made to the sampling framework during the last three days of fieldwork, in order to ensure that the sample was representative of the entire population of silver surfers according to the different Spanish geographical areas. This avoided the over-representation of some autonomous regions such as Madrid, Catalonia, and Andalusia, although these regions have the greatest presence in the sample simply because they have the largest populations in Spain.

The data were collected from 4–12 February of 2019 from the different Spanish territories by a national marketing company that conducted the survey by telephone in order to ensure that senior internet users with fewer online skills were able to complete the survey satisfactorily. This telephone approach enabled access to a broader spectrum of participants aged 60 and over, ranging from the most basic users of new technologies to the most advanced. Thus, it reflects a truer picture of this population group than would have been the case if the survey had been conducted with a senior online panel, which would have created a potential bias toward more advanced internet users.

### Measurements and variables

Apart from the socio-demographic variables that some classifications of older internet users have employed in defining the different profiles (Gallistl and Nimrod, 2020; Nimrod, 2017; Van-Deursen and Helsper, 2015a), the survey in this study was designed bearing in mind that the purposes for which people use the Internet and their digital skills are more decisive than their socio-economic or socio-cultural levels with regard to achieving the benefits associated with such use (Van-Deursen and Helsper, 2018).

The design of the survey was based on previous scientific literature and four focus groups, which were carried out in previous phases of the projects within which the present study is included. The survey was structured in three thematic blocks, foreseeing four developmental paths according to the respondents’ experiences with e-commerce (Viñarás-Abad et al. 2022) and e-government (Sánchez-Valle et al. 2022), as these online activities involve greater difficulty (Van-Deursen and Helsper, 2015). Moreover, they were more significant in defining the profiles of the most advanced silver surfers in the proposed typology.

The first block of questions in the survey is related to variables that have enabled the measurement of the ways in which older Spanish adults use the Internet and ICT. Among the online activities identified by Van-Deursen and Van-Dijk (2014) in their use matrix, the study herein has taken into account some of those that are classified as commercial transactions, social interaction, information searches, novelties, and leisure. Moreover, the digital uses included in the survey involve the following diverse internet skills (Van-Deursen and Van-Dijk, 2009, 2011):

1. WhatsApp use: The growing importance of this application among older adults has prompted research focused on this trend (Fernández-Ardèvol and Rosales, 2018; Rosales and Fernández-Ardèvol, 2016), and has led Gallistl and Nimrod (2020) to consider this factor in their classification of silver surfer profiles according to the way this group uses social media. However, the prominence attributed to WhatsApp by older users in the focus groups, who described it as one of the greatest advances in technology for online communication with their loved ones, along with the fact that they did not identify it as a Social Network Site (SNS), prompted the inclusion of its use in the questionnaire separately from other social media.
2. Participation in social networks: Although the focus groups pointed to a widespread lack of interest among older adults in social media, the importance that SNSs have acquired, together with their consolidation as a discriminating factor in the development of typologies of senior internet users (Elueze and Quan-Haase, 2018; Gallistl and Nimrod, 2020; Mason and Pereira, 2011; Nimrod, 2017; Quan-Haase et al. 2018; Vulpe and Crăciun, 2020), led to their inclusion in this study. Nevertheless, older people tend to prefer traditional and synchronous forms of communication (Nimrod, 2017). Consequently, their involvement in Social Network Sites (SNSs) is low, as these sites are generally used by digitally-advanced profiles (Quan-Haase et al. 2018) who have a predilection for online leisure tasks (Entertainment Seekers) (Gallistl and Nimrod, 2020). Conversely, silver surfers who feel a strong rejection of technology have no interest in participating in social media (Mason and Pereira, 2011).
3. Checking e-mail and/or reading digital news: Using e-mail is one of the most practical and easy-to-use online activities, which has become widespread among older internet users (Elueze and Quan-Haase, 2018; Mason and Pereira, 2011; Quan-Haase et al. 2018; Vulpe and Crăciun, 2020). Furthermore, reading the news in digital media is part of the usual pattern of online consumption among internet users over 60 (Gallistl and Nimrod, 2020; Mason and Pereira, 2011; Nimrod, 2017; Van-Deursen and Helsper, 2015a). As a result, it was considered appropriate to include a category in the survey that would combine these two traditional uses that are so widespread among internet users over 60, in order to verify whether it is still a determining variable in the definition of different clusters of silver surfers.
4. Searching for information on the Internet (price comparisons, health, weather, etc.): The use of search engines to find information in the digital realm has also been identified as a relatively simple online activity for senior internet users (Quan-Haase et al 2018; Van-Deursen and Helsper, 2015a), whose competence in this area improves depending on their frequency of daily use (Loos, 2011). However, Mason and Pereira (2011) have revealed the diversity of profiles among older internet users in terms of their information-seeking behaviour on the Web. It is therefore important to consider this use of the Internet in developing a taxonomy of silver surfers.
5. Use of electronic banking: Online banking has often been associated with the profiles of older internet users who are more advanced (Quan-Haase et al. 2018) and practical (Mason and Pereira, 2011). In general, silver surfers show a high level of scepticism toward online banking, due to concerns about potential fraudsters accessing their personal data when seniors carry out bank transactions (Elueze and Quan-Haase, 2018). The focus group participants confirmed this apprehension, pointing to the difficulties they have experienced as a result of the digital transition of Spanish banking, which has forced them to carry out tasks with which they are unfamiliar. Moreover, they feel uncomfortable and insecure in doing so. Therefore, this is considered a relevant factor in identifying those profiles that will have greater difficulty in achieving full digital inclusion.
6. Use of e-commerce: Online shopping is a task that also appears to be associated with highly pragmatic older surfers, and it tends to generate a lot of suspicion among the senior population as a whole with regard to the protection of their personal data (Elueze and Quan-Haase, 2018; Mason and Pereira, 2011; Viñarás-Abad et al. 2022). However, the convenience and cost savings offered by e-commerce, as confirmed by several focus group participants, could be a determining factor in differentiating between groups of silver surfers, especially the younger profiles (Van-Deursen and Helsper, 2015a).
7. Taking advantage of e-government, or online government administrative procedures: Although many older internet users consider the websites of public administrations and governments trustworthy (Mason and Pereira, 2011; Sánchez-Valle et al. 2022), several focus group participants did not feel safe when navigating these sites. In this regard, within the group known as Cynical Experts, Elueze and Quan-Haase (2018) note that some older adults are concerned about government surveillance in the digital realm. Furthermore, in a brief analysis of internet use by older adults for online administrative tasks, according to Van-Deursen and Helsper (2015a), the silver surfer profile that makes the best use of public services is more experienced, male, and younger. This includes online administrative tasks carried out at various levels (municipal, regional, and national), public services (health, leisure, welfare), and citizen obligation (various regional taxes). Thus, the range of procedures involving administration has become broader than in previous research.

The second block of questions in the survey is related to the digital abilities, skills, and capacities of internet users over 60 years of age, paying special attention to two factors:

1. Users’ self-perception of their own level of digital knowledge for making online purchases and carrying out public administration tasks on the Internet.
2. The level of digital knowledge considered necessary by the respondents to adequately carry out these online activities (e-commerce and e-government).

To measure both levels, a scale of four options (Low, Average, High, and Very High) was used in order to avoid allowing respondents to take refuge in the middle ground within a range of five choices, and to ensure that they clearly stated the level of digital knowledge they believe they have, as well as the level they consider necessary for carrying out such online activities. The need to include these variables is justified either by discrepancies between findings from previous studies of silver surfers regarding their use of e-commerce (Elueze and Quan-Haase, 2018; Mason and Pereira, 2011; Van-Deursen and Helsper, 2015a; Viñarás-Abad et al. 2022), or by the lack of more precise knowledge about the way this social group uses online procedures with public administration beyond civil services (Van-Deursen and Helsper, 2015a; Sánchez-Valle et al. 2022). Furthermore, although self-recognition of one’s level of technological skill is not always an indicator of a specific use of e-commerce or e-government, this self-perception can act either as an incentive or a disincentive among some older adults, as acknowledged by some of the focus group participants. Quan-Haase et al. (2018) have found that many older adults do not allow their level of digital knowledge to influence their online participation. In fact, some seniors engage in a wide range of digital activities despite acknowledging only a basic level of technological competence. Nevertheless, online tasks that involve more relevant and indirect benefits in the offline lives of silver surfers (e-commerce, e-government, and public services) require more digital experience and tend to be more efficiently exploited by those with a higher level of education (Van-Deursen and Helsper, 2015a, 2015b). On the other hand, it has been observed that seniors tend to judge themselves harshly in digital matters when compared to younger, more highly skilled family members, despite mastering several online activities (Quan-Haase et al. 2018), which is something that might negatively influence the self-perception of their digital knowledge.

The third and final block of questions in the survey measures variables related to the level of trust in e-commerce platforms and e-government websites by this group. In general, a lack of trust in the Internet has led some older adults to deliberately restrict their use of technology, such as the Rejecters (Mason and Pereira, 2011), or has prevented them from experimenting with new online activities, as in the case of Reluctant Users (Quan-Haase et al. 2018). The attitudes and concerns of older adults about their privacy in the digital sphere may heighten fears that could prompt them to limit their use of activities that require the disclosure of certain data, such as online banking (Intense Pragmatists, Cynical Experts), or online shopping (Fundamentalists) (Elueze and Quan-Haase, 2018). While a certain number of silver surfers consider online government websites to be safe and trustworthy (Mason and Pereira, 2011), other more digitally-advanced profiles (Cynical Experts) are concerned about government surveillance (Elueze and Quan-Haase, 2018). The participants of the focus groups highlighted the mistrust felt by older surfers toward e-commerce and e-government, while acknowledging the prominence these activities are gaining in Spanish society as a result of the digital transition. Trust in online shopping and administrative procedures are likely to be determining variables in the definition of different types of silver surfers. For this reason, the survey includes questions that measure the trust of older adults through the use of items related to their self-perception of internet security when performing online administrative tasks or purchases. Regarding these issues, the respondents were asked to respond to the following questions:

1. Have you dared to make a first purchase online or to carry out a public administration procedure through the Internet without asking for help? This was a dichotomous Yes/No question.
2. Do you feel confident shopping online or carrying out procedures with public authorities on the Internet? This question allowed three possible answers: Yes/No/Sometimes.
3. How do you rate your level of trust in e-commerce platforms? To measure their degree of trust in e-commerce sites, a scale of four possible answers was offered (Low, Average, High, and Very High), again to prevent doubtful respondents from taking refuge in the middle ground of a five-choice scale.
4. How do you rate your level of trust in public administration? Following the reasoning in the previous question, the same four-choice scale was offered (Low, Average, High and, Very High).

Furthermore, the survey design estimated four paths according to the respondents’ acknowledged experience with online shopping and administrative procedures:

1. Path 1 (*N* = 160; 39.5%): This refers to the specific questionnaire completed by respondents in the sample who shop and carry out administrative tasks online.
2. Path 2 (*N* = 84; 20.7%): This is the set of questions specifically answered by survey participants who shop online but do not carry out online administrative tasks.
3. Path 3 (*N* *=* 71; 17.5%): This is the path followed by respondents in the sample who do not shop online but do carry out digital administrative procedures.
4. Path 4 (*N* = 90; 22.5%): This is the block of questions answered by respondents in the sample who do not make purchases through the Internet nor carry out online administrative procedures.

The questions in the first block of the survey acted as a filter to guide the respondents toward one of the four paths so they could complete the survey according to the specific ways they use ICT. The estimation of the time needed to complete the survey was approximately 18 min, slightly less for Path 4.

### Data analysis

The main objective of this study is to identify different profiles of silver surfers according to the ways they use the Internet. This involves the branch of statistics known as unsupervised learning, whereby a set of methods and algorithms is used to identify structures and similarities when there is no response variable. In other words, although there is no reference variable to study, this paper has investigated the common features of observation using cluster analysis (Kaufmann and Rousseeuw, 1990).

As is customary in data analysis, the first task is to load and pre-process the dataset to ensure it is formatted properly in order to apply the subsequent algorithms (Zamora-Saiz et al. 2020). When performing an analysis based on clustering methods, three main steps should be conducted; firstly, calculate the dissimilarity matrix; secondly, apply the actual clustering method to the matrix; and thirdly, validate the results (James et al. 2013). Throughout these steps, several a priori choices arise that must be decided upon by the analyst. It is important to emphasise that all these decisions have an impact on the final result. Consequently, validation is highly important.

Validation and coherence with real life enable meaningful results, which is the best way to make the most suitable selections based on the following:

1. Distance: Which separation space shall be used to set up the dissimilarity matrix?
2. Clustering method: Which method shall be used to find the similarities within the matrix, and if that method has specific parameters, which parameters will be chosen?
3. Number of clusters: How many groups offer the best balance between the performance of each category and the description of the subjects in each cluster.
4. Validation measures: Other measures of quality must be used to assess performance with an unsupervised learning method where there is no reference variable. Moreover, the importance given to one or another of these measures will be decisive (Fernández-Santana, 1991).

All the statistical calculations were performed using R 4.0.2 under the front end RStudio 1.3.1056. Even though only the most relevant clustering is presented herein, numerous combinations of the previously mentioned choices were tested. Other combinations produced similar results, thereby increasing confidence in the findings; if several algorithms arrive at similar results, it is likely that a ground truth has been revealed.

Regarding the dissimilarity matrix, several distances were considered^1^, and optimal results were achieved through the KODAMA algorithm^2^ using k-nearest neighbours with *k* = 7 (Cacciatore et al. 2017).

Afterward, K-means clustering was selected as the most suitable option for carrying out the study using seven clusters. Selecting this algorithm and the number of clusters was due to both internal^3^ and stability measures. Internal measures were used to verify whether the observations in each group were similar and if the groups were separated; stability measures were employed to check the consistency and strength of the results^4^. Both internal and stability measures were implemented using clValid (Brock et al. 2008).

Even though the clustering results might be considered remarkable, this procedure acts as a black box. It detects similarities between observations, and it might even reveal the depiction of certain social behaviours, yet the way in which the selection had been reached would not be clear. In order to counteract this factor, the variables that were key in identifying the differences between the groups were extracted and ranked. Additionally, bi-dimensional plots of the clusters have been provided.

## Results

### Digital skills and internet use by older people (SO1 and RQ1)

This section presents the results of the survey and a brief interpretation of the data. The findings outline the characteristics of Spanish silver surfers according to a specific set of internet uses, digital skills, and trust in e-commerce and e-government, highlighting their lack of homogeneity.

Table 1 shows the main digital activities carried out by silver surfers. In general, it reveals that the use of WhatsApp is the most common (91.4%), followed by reading the news and checking e-mail (83.0%). More than half of the respondents perform searches on the Internet, compare the prices of products and services (57.3%), shop online (60.2%), and carry out online tasks with public administration (57.0%). Other more advanced uses, such as social media (47.9%) and online banking (43.5%), are performed by less than half of the internet users. In analysing the breakdown by path, Table 1 shows that respondents in Path 1 are the most versatile silver surfers in terms of internet use. However, they have the lowest percentage of internet use for online searches, as only 43.8% of them carry out this activity, despite the fact that they all use e-commerce and perform online administrative procedures. Moreover, these users reach the highest percentages among the rest of the digital uses tested (WhatsApp, e-mail/news, social media, and online banking). Respondents in Path 4 are older adults who are the most restrictive in their use of the Internet and digital media, as they carry out neither online shopping nor administrative procedures, and they also have the lowest percentages in all of the uses evaluated, except for consulting e-mail and reading online news (lower among respondents in Path 3) and participation in social media (lower among respondents in Path 2) (Table 1).

**Table 1:** Overall use of the Internet by silver surfers according to the response path (*N* = 405).

	WhatsApp	Social Media	Mail/ News	Searches (information)	Online banking	Online shopping	Online administration (admin.) tasks
Path 1	95.0	56.3	86.9	43.8	52.5	100.0	100.0
Path 2	89.3	39.3	81.0	69.0	40.5	100.0	0.0
Path 3	91.5	47.9	76.1	70.4	36.6	0.0	100.0
Path 4	86.7	41.1	83.3	60.0	35.6	0.0	0.0
Total	91.4	47.9	83.0	57.3	43.5	60.2	57.0

Data provided in percentages.

With regard to the self-perceived level of skill in using the Internet by those surveyed, the data in Table 2 indicate that 53.1% believe they have an average level, and only 22.0% consider it to be high or very high, the latter of which is similar to the figure for those who believe they have a low level (24.9%). Regarding the level considered necessary for adequate internet use, nearly half (47.4%) believe that an average level is sufficient; 33.8% state that a high or very high level is required; and 18.8% consider that a low level of skill is enough.

**Table 2:** Level of digital skills that silver surfers perceive they have according to the path response, displayed cumulatively (*N* = 405).

		Path 1	Path 2	Path 3	Path 4	Total
Low	Digital skills level	11.3	23.8	29.6	46.7	24.9
	Digital skills level for practical use	11.9	19.0	19.7	30.0	18.8
Average	Digital skills level	53.1	60.7	53.5	45.6	53.1
	Digital skills level for practical use	42.5	56.0	54.9	42.2	47.4
High	Digital skills level	28.1	14.3	15.5	4.4	17.8
	Digital skills level for practical use	34.4	21.4	21.1	23.3	26.9
Very high	Digital skills level	7.5	1.2	1.4	3.3	4.2
	Digital skills level for practical use	11.3	3.6	4.2	4.4	6.9

Data provided in percentages.

The breakdown of the data by response path regarding both the level of self-perceived digital skills of the silver surfers, and the degree of technological knowledge they consider necessary to make beneficial use of the Internet and digital media, show the following: Although the respondents in Path 1 are the most versatile in their use of the Internet, only 7.5% believe they have a very high level of digital skill. On the other hand, respondents who admit to engaging in a much more restricted use of online activities (Path 4) generally see themselves as having a low level of digital skills (46.7%). However, 3.3% consider that they have a very high level of digital knowledge, and up to 30% say that a low level of this type of skill is enough for the purpose of using the Internet satisfactorily. An average level of self-perceived digital skills of the respondents is the most widespread among those who completed Path 1 (53.1%), Path 2 (60.7%), and Path 3 (53.5%). On the other hand, an average level is also what is considered necessary for the successful or satisfactory use of digital media, according to the majority of the respondents in all the Paths (1–42.5%, 2–56.0%, 3–54.9%, and 4–42.2%) (Table 2).

Trust in e-commerce platforms and e-government websites is very similar. As shown in Table 3, 43.0% claim to have an average level of trust in e-commerce, which increases to 48.9% for e-government. The results among those with a low level of trust are also close, although e-commerce shows a higher percentage of low trust (24.7%) than e-government (21.7%). Likewise, similar overall percentages are found for high and very high levels of trust in e-commerce (32.3%) and e-government (29.4%) (Table 3). Segregating the results by response path, it can be seen that an average level of trust in e-government is the most widespread among all survey participants, even among respondents in Path 4, who make neither online purchases nor carry out administrative tasks through the Internet. However, this is not the case for e-commerce. While it reaches a mostly average level of trust among respondents in Path 1 (43.1%) and Path 2 (53.6%), it elicits low levels of trust predominantly among participants who completed Path 3 (56.3%) and Path 4 (43.3%). Silver surfers who use the Internet in more diversified ways (Path 1) have the highest percentages in the categories of high and very high levels of trust in both e-commerce (41.3%; 7.5%) and e-government (36.9%; 8.8%) (Table 3).

**Table 3:** Overall level of trust that silver surfers perceive they have regarding e-commerce and e-government according to the path response (*N* = 405).

		Path 1	Path 2	Path 3	Path 4	Total
Low	Trust in e-commerce	8.1	9.5	56.3	43.3	24.7
	Trust in e-government	11.3	27.4	14.1	41.1	21.7
Average	Trust in e-commerce	43.1	53.6	31.0	42.2	43.0
	Trust in e-government	43.1	53.6	62.0	44.4	48.9
High	Trust in e-commerce	41.3	32.1	11.3	14.4	28.1
	Trust in e-government	36.9	17.9	18.3	12.2	24.2
Very high	Trust in e-commerce	7.5	4.8	1.4	0.0	4.2
	Trust in e-government	8.8	1.2	5.6	2.2	5.2

Data provided in percentages.

Regarding the feeling of internet security among older adults for shopping and carrying out administrative procedures, 69.6% of those surveyed have at some point performed an administrative task without assistance, while 66.2% feel safe shopping online and carrying out administrative procedures. The breakdown of the data reveals that a high percentage of respondents who completed Path 1 (78.1%), Path 2 (78.6%) and Path 3 (76.1%) say that they trust the digital environment for carrying out e-commerce and e-government activities. In the same vein, these silver surfers are very willing to make online purchases or perform administrative tasks online without help, especially those who responded to the survey in Path 1 (83.1%) and Path 2 (81.0%). On the other hand, as the respondents in Path 4 are much more limited in carrying out online tasks, most of them admitted not feeling safe in carrying out e-commerce and e-government activities (61.1%). A high percentage of this group of participants (65.6%) also admitted not having dared to make purchases or perform administrative procedures online, which reflects their insecurity when using digital media for certain tasks (Table 4).

**Table 4:** Overall security perceived by silver surfers when making online purchases and performing administrative procedures through the Internet, according to the response path (*N* = 405).

		Path 1	Path 2	Path 3	Path 4	Total
Yes	Perform online administrative procedures or purchases without assistance	83.1	81.0	70.4	34.4	69.6
Yes	Internet security perceived when shopping or carrying out administrative tasks online	78.1	78.6	76.1	25.6	66.2
No	Perform online administrative procedures or purchases without assistance	16.9	19.0	29.6	65.6	30.4
No	Internet security perceived when shopping or carrying out administrative tasks online	19.4	11.9	15.5	61.1	26.4
Sometimes	Internet security perceived when shopping or carrying out administrative tasks online	2.5	9.5	8.5	13.3	7.4

Data provided in percentages.

These descriptive data point to differences among internet users over 60 years of age in using the Web, their level of digital skill, and their degree of trust in digital media for carrying out certain online tasks. As such, different profiles are presumed to exist, and defining them requires a cluster analysis that will allow older internet users to be grouped according to these characteristics in order to identify those who are most vulnerable.

### Description of silver surfer groups (SO2 and RQ2)

In Tables 5 and 6, the responses that best represent each of the groups can be observed. This is not to say that all subjects in the same group responded in exactly the same way, but that the responses were similar. At the same time, in all cases the responses were sufficiently different from those of the other groups, which has enabled their differentiation.

**Table 5:** Characteristics of the different profiles regarding general use of the Internet by silver surfers (*N* = 405).

Cluster	WhatsApp	Social Media	Mail/ News	Searches (information)	Online banking	Online shopping	Online admin. tasks	*N*
Able	1. Yes	1. Yes	1. Yes	1. Yes	0. No	1. Yes	1. Yes	98
Daring	1. Yes	1. Yes	1. Yes	0. No	1. Yes	1. Yes	1. Yes	95
Sceptical	1. Yes	0. No	1. Yes	0. No	0. No	0. No	0. No	72
Novice	1. Yes	0. No	1. Yes	1. Yes	0. No	1. Yes	0. No	53
Confident	1. Yes	0. No	0. No	1. Yes	0. No	1. Yes	1. Yes	52
Insecure	0. No	0. No	1. Yes	1. Yes	0. No	0. No	0. No	19
Practical	0. No	0. No	1. Yes	1. Yes	1. Yes	1. Yes	1. Yes	16

KODAMA algorithm.

**Table 6:** Characteristics of the different profiles with regard to level of expertise, internet security, and the self-perception of trust by silver surfers (*N* = 405).

Cluster	Digital skills level	Digital skills level for adequate use	Perform online admin. procedure/purchase without assistance	Internet security	Trust in e-commerce	Trust in e-government	*N*
Able	2. Average	2. Average	1. Yes	2. Yes	2. Average	2. Average	98
Daring	2. Average	3. High	1. Yes	2. Yes	3. High	3. High	95
Sceptical	1. Low	2. Average	0. No	0. No	1. Low	2. Average	72
Novice	2. Average	2. Average	1. Yes	2. Yes	2. Average	2. Average	53
Confident	2. Average	2. Average	1. Yes	2. Yes	2. Average	2. Average	52
Insecure	1. Low	2. Average	0. No	0. No	1. Low	1. Low	19
Practical	2. Average	3. High	1. Yes	2. Yes	3. High	2. Average	16

KODAMA algorithm.

The following typology of silver surfers was established, with seven groups identified (Tables 3 and 4):

1. Able (Tables 5 and 6): This cluster has the highest number of seniors in this study who are internet users, with a total of 98 individuals. It reflects the pattern of a user who is able to perform all the activities in question with the exception of online banking. They believe they have an average level of internet skill, which they consider sufficient for using the Internet adequately. This group feels safe using the Web, and they display an average level of trust in both e-commerce and digital administration.
2. Daring (Tables 5 and 6): This group is the second most numerous with 95 individuals. The profile of this user is a person with an advanced digital skills level who benefits from the Internet. This user claims to utilise all the services provided by the Web, except for information searches. However, this type of user also perceives that their internet skills are only average and that a high level is necessary in order to properly carry out certain online tasks, such as shopping and administrative procedures. This group displays a high level of trust in e-commerce and e-government, and feels safe when using the Internet.
3. Sceptical (Tables 5 and 6): This cluster is third in terms of the number of individuals, with a total de 72. This profile is sceptical about internet use. Using WhatsApp and checking their e-mail are the only activities they perform. They claim to have a low level of internet skill and that an average level is required in order to use the Web correctly. This type of user does not dare perform online shopping or electronic administrative tasks alone. Moreover, this group feels unsafe when using the Internet and displays a low level of trust in e-commerce platforms, although the level increases to average with regard to e-government websites.
4. Novice (Tables 5 and 6): This is the fourth most numerous group, with 53 internet users. The pattern of online behaviour for these individuals is to consult WhatsApp, search for information, check their e-mail, and read the news. As for more advanced uses, they claim to engage in online shopping, but not social media, online banking, nor electronic administration. They also claim to have an average level of internet skill, which is perceived as being adequate for using the Web. This group feels safe using the Internet and displays an average level of trust both in e-commerce and e-government.
5. Confident (Tables 5 and 6): This is the fifth cluster by size, consisting of 52 individuals. This profile uses WhatsApp, performs internet searches, shops online, and performs electronic administrative tasks. They claim to have average internet skills, which they consider suitable for administrative procedures and other internet operations. This type of user is not afraid to shop online and perform administrative tasks without assistance, and they feel safe in doing so. They claim to have an average level of trust in e-commerce and e-government platforms.
6. Insecure (Tables 5 and 6): This group consists of 19 individuals, making it the sixth cluster in terms of numbers. The profile of this group is that of a basic internet user who performs simple activities, such as checking e-mail, reading the news, and searching for various kinds of information on the Web. This profile does not use advanced electronic services such as online shopping or digital administration. It is the only group that does not dare to shop or perform administrative tasks online without assistance. They claim to have a low level of internet skill, yet they say an average level is necessary in order to take full advantage of the Web. This cluster displays minimal trust in e-commerce and e-government websites, as well as a low level of trust in internet security.
7. Practical (Tables 5 and 6): This cluster consists of 16 individuals, which makes it the least numerous of the seven groups identified. This profile performs all the activities in question, with the exception of those of a more communicative nature, such as WhatsApp and social media. They claim to have an average digital skills level, yet consider that a high level is necessary in order to take full advantage of the opportunities provided by the Web. They feel safe when using the Internet and have a high degree of trust in e-commerce websites, although this trust decreases to an average level in terms of administrative websites.

**Fig. 1:**
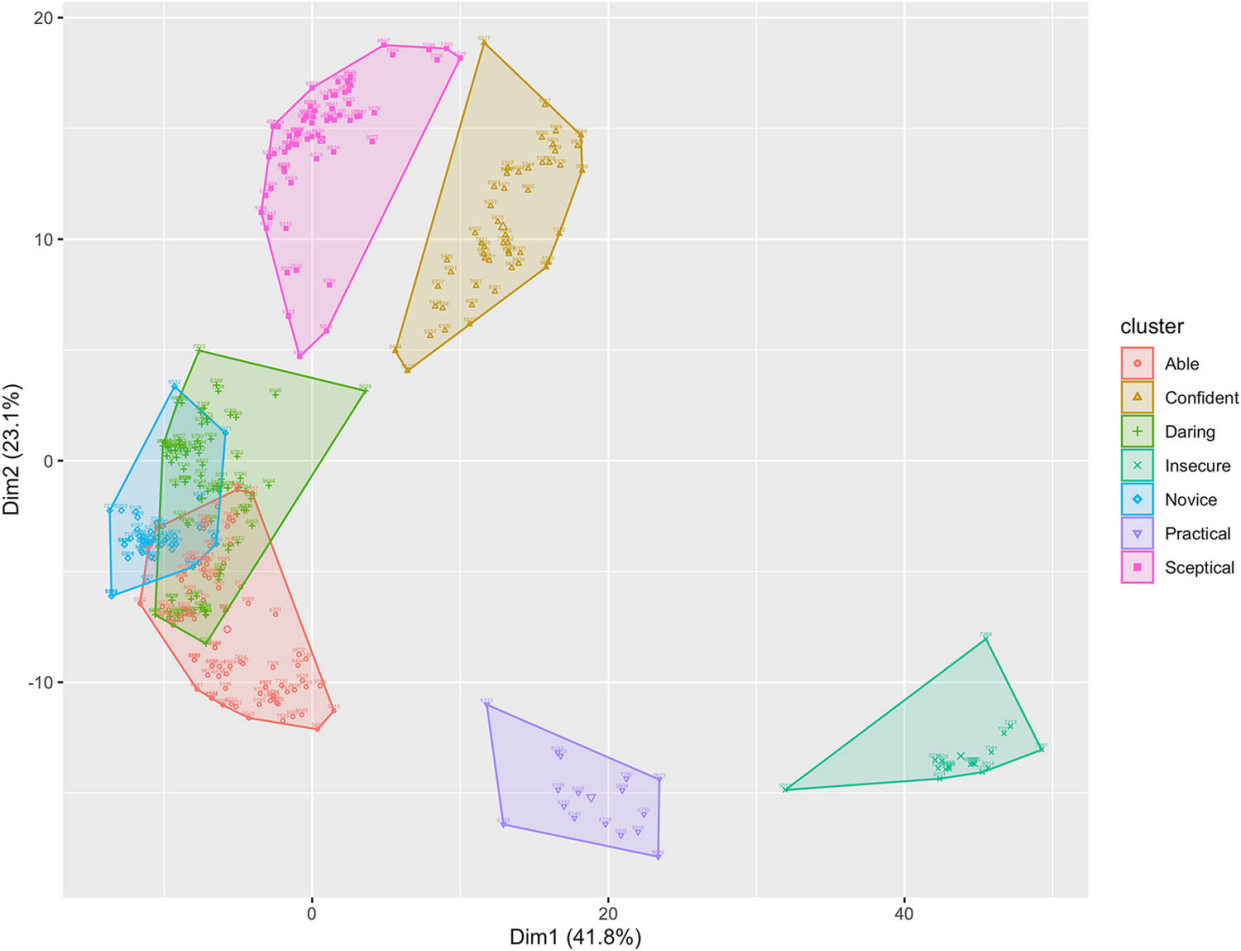
Graphic representation of the clusters of silver surfers. Created using R.

A cluster analysis was conducted, along with a prior dimensionality reduction stage, as described in the methodology section. These techniques have enabled the selection of k-means as the optimal clustering method, or in other words, as the best trade-off between internal and stability measures. K-means clustering has been used with the seven groups, as shown in Fig. 1.

**Fig. 2:**
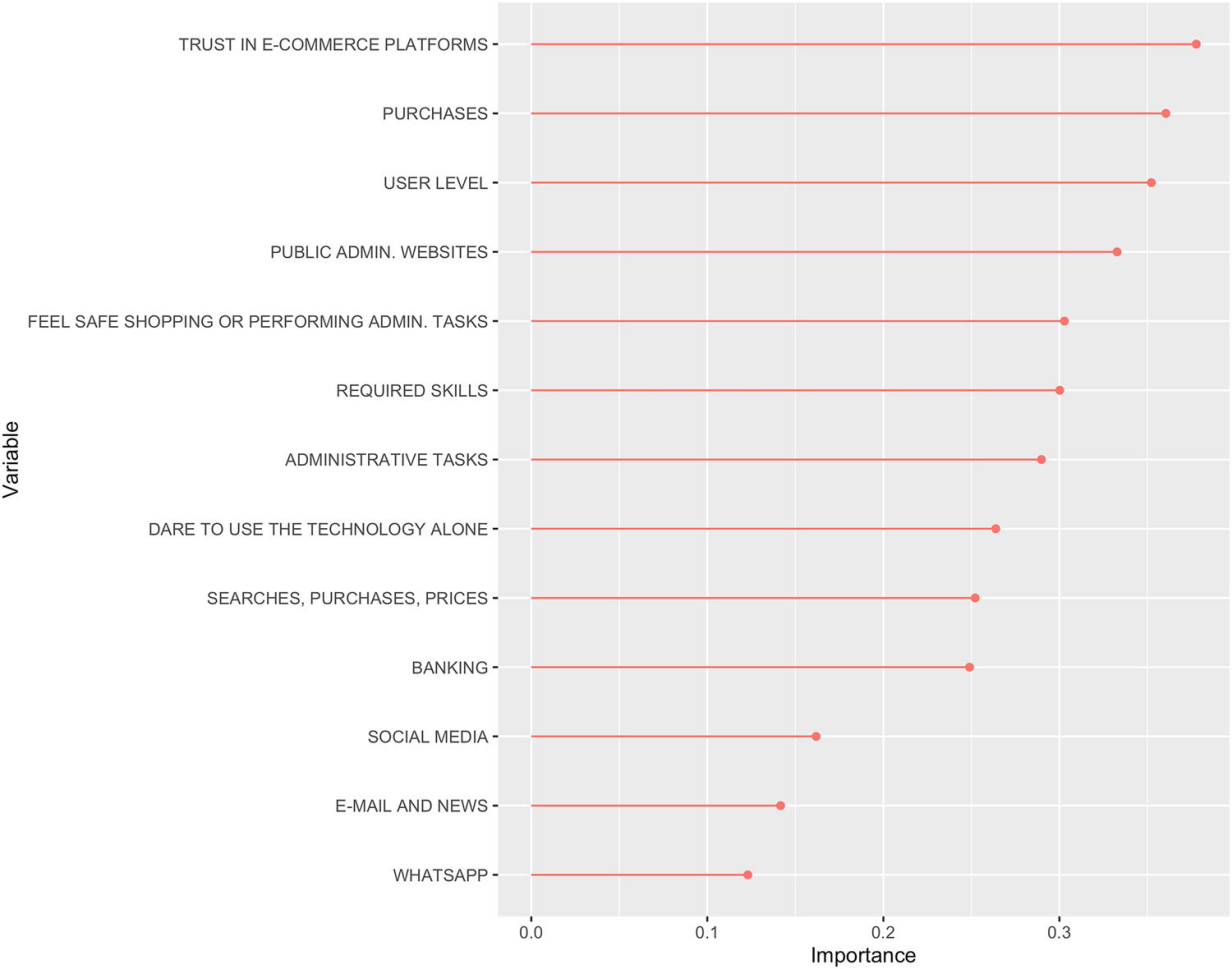
Importance of the responses provided in creating a classification of the groups. Created using R.

In Fig. 2, the responses that achieved the highest level of importance, or weighting, in developing a classification of the groups can be observed. These refer to questions related to the level of trust in e-commerce, online shopping, the perceived skill level of the user, and with less importance, the level of trust in e-government websites, as well as a feeling of internet security when performing purchases or e-government procedures online. Consequently, these variables determine the major differences between the groups identified in the analysis. The variables with less impact on the groupings are those related to the use of WhatsApp, checking e-mail, reading online news, the use of social media, and online banking. With regard to these aspects, the responses of all the participants are highly similar, with the first three standing out for their extensive use, and the last one for its low level of use.

### Characteristics of the most insecure and least skilled silver surfers (SO3 and RQ3)

According to the cluster analysis, the Sceptical and Insecure are the silver surfers who display a low level of digital skills and greater mistrust of the Internet for certain online activities. These profiles of senior navigators are the ones who mostly completed Path 4 of the survey.

Tables 7 and 8 show the socio-demographic characteristics of the participants in the study according to the response path they completed. It can be observed that the respondents of Path 4 are very balanced in terms of gender, while more than half of the subjects who completed the remaining paths are men, especially those who answered the survey through Path 3 (Table 7). The average age of the respondents to Path 4 (69.9 years) is more than two years older than the average age of the participants who completed Path 1 (67.7 years), yet there are no significant differences with regard to the respondents to Path 2 (69.5 years) nor Path 3 (69.1 years) (Table 7).

**Table 7:** Overall socio-demographic profile of survey participants according to the response paths (*N* = 405).

Path 1		Path 2		Path 3		Path 4		Total	
Gender	Age	Gender	Age	Gender	Age	Gender	Age	Gender	Age
M = 58.1	68.8	M = 59.5	69.9	M = 69.0	69.9	M = 51.1	71.4	M = 58.8	69.8
W = 41.9	66.2	W = 40.5	68.9	W = 31.0	67.3	W = 48.9	68.4	W = 41.2	67.5
*N* = 160	67.7	*N* = 84	69.5	*N* = 71	69.1	*N* = 90	69.9	*N* = 405	68.8

Data provided in percentages.

**Table 8:** Place of residence of survey participants according to the response paths (*N* = 405).

Autonomous Region	Path 1	Path 2	Path 3	Path 4
Andalusia	21.3 (34)	16.7 (14)	16.9 (12)	18.9 (17)
Catalonia	16.9 (27)	23.8 (20)	15.5 (11)	28.9 (26)
Madrid	20.6 (33)	23.8 (20)	22.5 (16)	14.4 (13)

Data provided inpercentages.

Moreover, no significant disparities were detected in terms of place of residence, as the highest percentage of respondents in each path reside in Spain’s most populated Autonomous Regions. However, it is surprising that the respondents in Path 1, who make more diverse and intense use of the Internet, live in Andalusia, as this region is traditionally considered to be less developed than Catalonia or the Autonomous Region of Madrid. At the same time, the majority of the respondents in Path 4, who make more limited use of digital media, live in Catalonia, which is historically considered the most advanced Autonomous Region in Spain in socio-economic and digital terms (Table 8).

The results of the cluster analysis enabled these data to be refined based on the socio-demographic characteristics of each group of silver surfers. Sceptical and Insecure users have a more limited range of online activities, foregoing the indirect benefits of social media, e-commerce, and e-government, yet their socio-demographic characteristics are similar to those of other internet users who are older. According to the data presented in Table 9, as far as gender is concerned, the most notable differences are perceived in the Insecure, Practical, and Able, with a predominantly male profile. Women are not in the majority in any of the profiles of silver surfers, which suggests that the Internet continues to be male-dominated, although it is evenly distributed in the Sceptical cluster, and nearly balanced among the Confident, Novice, and Daring clusters. Age is not a discriminating variable among the groups identified, nor does it seem to be a major determining factor among the study participants in defining their level of digital skills and/or assortment of internet uses. Thus, the Practical category has the highest average age (70.3 years), although these silver surfers use the Internet in a diversified way, including tasks that require more digital experience and confidence, yet are very practical for them (online banking, e-commerce, and e-government). The Sceptical and Novice (69.9 years on average), and Insecure (69.2 years on average), are the next oldest silver surfers, while the Daring are the youngest (68 years on average). Therefore, no significant differences can be drawn, which indicates that age does not appear to be a factor that influences silver surfers’ digital knowledge and the assortment of specific ways they use the Internet.

**Table 9:** Socio-demographic profile of each cluster of silver surfers (*N* = 405).

	Able	Daring	Sceptical	Novice	Confident	Insecure	Practical
**Gender**							
Men	62.20	55.80	50.00	54.70	53.80	89.50	87.50
Women	37.80	44.20	50.00	45.30	46.20	10.50	12.50
**Age**							
Median	69	68	71	71	68.5	70	70
Mean	68.5	67.9	69.9	69.9	68.2	69.2	70.3
SD	4.9	4.8	4.5	4.7	4.9	4.4	3.6
Min.	61	61	61	61	61	61	63
Max.	77	77	77	77	77	76	76
**Autonomous Region**							
Madrid	18.37	21.05	20.83	16.98	23.08	15.79	31.25
Catalonia	12.24	15.79	20.83	33.96	30.77	26.32	6.25
Andalusia	16.33	24.21	18.06	15.09	19.23	15.79	25.00

KODAMA algorithm.

In terms of place of residence, most silver surfer clusters are distributed according to the Spanish population density. Thus, the Autonomous Region of Madrid is where the highest percentage of the Able and Practical reside; Catalonia is the region with the highest percentage of Novice, Confident, and Insecure; and Andalusia has the highest percentage of Daring. The highest percentage of the Sceptical group has been registered in both Madrid and Catalonia. Probably the most unexpected result is that Catalonia has the lowest percentage of Practical out of the three Spanish regions, as it is traditionally classified as the most technologically developed autonomous region in all of Spain (Table 9).

To enable the digital inclusion of the Sceptical and Insecure so they can take advantage of the indirect benefits of internet use at the social, cultural, economic, and personal levels, intervention policies should be developed to encourage them not only to use e-commerce and e-government, but social media and online banking as well. To do so, it appears to be crucial to promote their level of trust in the Internet for the development of all these activities. It is essential to highlight the importance of designing and implementing these kinds of measures, as this paper shows that 17.8% of silver surfers are Sceptical, which makes this profile the third most numerous group among Spanish internet users aged 60 and over.

## Conclusions and discussion

### Conclusions

Silver surfers behave in a variety of ways on the Internet, displaying various levels of expertise in performing online tasks, thereby generating a certain degree of inequality with regard to social inclusion. The results obtained in this study have allowed differences to be identified among Spanish silver surfers according to the specific ways they use the Internet to carrying out certain online tasks, their level of skill, and their trust in online use. This has made it possible to achieve the main objective of the study, in addition to the first of the specific objectives (SO1), and provide an answer to RQ1 as well. According to the cluster analysis, the most basic uses of the Internet are shared by nearly all internet users aged 60 and over, yet not the more specific tasks. However, online shopping and administrative procedures are activities that are increasingly common and more frequent among this group, with the majority of respondents having shopped or carried out administrative tasks online without assistance (the Able, Daring, Novice, Confident, and Practical).

In total, up to seven different profiles of silver surfers have been identified, with contrasts in their digital use, level of digital skill, and trust in the Internet for making purchases and completing online administrative tasks, which allows RQ2 to be answered, along with the associated SO2. Furthermore, with regard to SO3 for the purpose of responding to RQ3, an in-depth analysis of the profiles provides a specific definition of those who are most distrustful and least digitally skilled (the Sceptical and Insecure), offering data that could help redefine intervention policies that might encourage their socio-digital inclusion, and consequently their successful ageing.

The two most widespread profiles of silver surfers in Spain carry out a wide variety of online tasks. The Able, who represent 24.2% of the sample, perform all the digital activities tested with the exception of online banking. The Daring (23.5% of the participants) use the Internet for all the uses analysed except online searches. The third most prevalent group of senior internet users in Spain are the Sceptical (17.8% of the sample); unlike the previous profiles, they have a much more limited variety of uses, thereby renouncing the opportunities offered by online banking, e-commerce, and e-government.

The level of trust in e-commerce and e-government is also an important variable in determining the different types of silver surfers. In general, the groups do not display a high degree of trust in e-commerce, except for the Daring and Practical. Specifically, the Daring and Practical are the only groups that use online banking. Furthermore, only the Daring express a high level of trust in electronic administration. Regarding internet user skills, the majority of the seniors who participated in this study do not consider themselves experts, although they believe that their level is sufficient to perform internet tasks that are both essential and desirable. The Insecure, Daring, Practical, and Sceptical perceive that a higher level of knowledge and expertise is necessary in order to use the Internet correctly, while the Confident, Novice, and Able claim to have an appropriate level for their needs and desired use of the Web. Thus, the self-defined profile of the user coincides with the desired level for adequate internet use, which is higher in the case of the most advanced (the Confident and Able) and lower in the case of those who perform only basic internet and ICT tasks (the Novice).

Despite the fact that none of the groups claim to have a high level of internet user skills, nearly all of them feel safe when navigating, except for the Insecure and Sceptical, who have low skill levels and do not dare shop or perform administrative procedures online by themselves. These users have the lowest level of skills, and therefore need incentives to expand their internet use in order to take advantage of the Web with the aim of gaining autonomy in the digital world.

Table 10 summarises the main characteristics of the seven clusters of silver surfers in Spain identified in this research.

**Table 10:** Silver surfer clusters in Spain (*N* = 405).

Profiles of silver surfers with an average level of digital knowledge who use the Internet in diverse ways		
Able (*N* = 98; 24.2%)	Daring (*N* = 95; 23.5%)	Practical (*N* = 16; 4.0%)
• Predominantly male.	• This profile is nearly equal between the genders.	• Male profile.
• They declare having an average level of digital skill, which they perceive as necessary for making satisfactory use of the Internet. They are not afraid to make purchases and carry out administrative tasks online without help. They believe the Internet is safe for online shopping and administrative procedures, which gives them an average level of trust, and they feel this is enough for carrying out these tasks.	• They report an average level of digital knowledge but consider that a high level of technical skill is necessary for making satisfactory use of the Internet. They have no problem carrying out purchases and administrative procedures online without help, as they consider the Internet to be safe for these purposes. They express a high level of trust in e-commerce and e-government.	• They recognise having an average level of digital skills but consider that a high level of digital knowledge is necessary in order to take advantage of the opportunities provided by the Internet. They shop and carry out administrative procedures online because they feel safe doing so, and they express a high level of trust in e-commerce and average trust in e-government.
• They participate in all the activities studied (WhatsApp, social media, the digital press, search engines, e-commerce, and e-government) with the exception of online banking.	• They make use of all the tasks analysed (WhatsApp, social media, e-mail, online press, digital banking, e-commerce, and e-government), except for search engines.	• They do not use the Internet socially, so they do not participate in social media nor WhatsApp. Their online behaviour is pragmatic (e-mail, reading news, search engines, online banking, e-commerce, and e-government.

Profiles of silver surfers with an average level of digital knowledge who make moderate use of the Internet	
Confident (*N* = 52; 12.8%)	Novice (*N* = 53; 13.1%)
• This profile is nearly equal between the genders.	• Profile nearly equal between the genders.
• They admit to having an average level of digital skills and believe this level is necessary in order to use the Internet productively. They have no objection to making purchases and carrying out administrative tasks online. Moreover, they feel safe when engaging in these activities in the digital sphere. They have an average level of trust in e-commerce and e-government.	• They believe they have an average level of technological skill and that such a level is necessary in order to make adequate use of the Internet. They make purchases and carry out administrative procedures online without help. They believe the Internet is a safe medium for carrying out these kinds of tasks. They have an average level of trust in e-commerce and e-government.
• They use WhatsApp, search engines, e-commerce, and e-government, but do not use social media, online banking, e-mail, nor the digital press.	• They use WhatsApp, e-mail, the online press, search engines, and e-commerce, but do not use social networks, online banking, nor e-government.

Profiles of silver surfers with a low level of digital knowledge and limited use of the Internet	
Sceptical (*N* = 72; 17.8%)	Insecure (*N* = 19; 4.7%)
• This profile is nearly equal between the genders.	• Predominantly male profile.
• They believe they have a low level of digital knowledge and that an average level is necessary in order to take full advantage of the Internet. They are afraid to make online purchases and carry out administrative procedures without help, as they feel very unsafe engaging in such activities. They admit to having a low level of trust in e-commerce, but an average level of trust in e-government.	• They perceive themselves as having a low level of digital knowledge and believe that an average level is necessary in order to make satisfactory use of the Internet. They are afraid to make purchases or perform administrative procedures online, as they do not feel safe in doing so, probably because their level of trust in e-commerce and e-government is low.
• Of all the uses analysed, they only use WhatsApp, e-mail, and the online press, yet they disregard the indirect benefits that other activities could offer them, such as online banking, e-commerce, e-government, social media, and search engines.	• They only use search engines, e-mail, and the online press. However, they fail to take advantage of the opportunities offered by the Internet for socialising (they do not use WhatsApp or social media), for obtaining economic benefits (they do not use online banking or e-commerce), or for personal gain that could offer convenience and save time (they do not use e-government).

## Discussion

Silver surfers display a growing interest in the Internet and ICT, recognising the opportunities it can provide to their everyday lives. Specifically, the communicative options offered by the Internet help seniors become more socially integrated and guarantee a healthy and active ageing process (Agudo-Prado et al. 2012), thereby optimising their quality of life from a psychological and socially-integrative point of view (Llorente-Barroso et al. 2015).

Previous studies have shown the negative impact of age on the level of people’s digital skills and their use of the new media (Friemel, 2016; Hargittai, 2002; Hunsaker and Hargittai, 2018; Olsson et al. 2019; Ragnedda et al. 2020; Van-Deursen and Helsper, 2015, 2018; Van-Deursen and Van-Dijk, 2019). These studies have confirmed inequalities that reflect heterogeneity among the population of older internet users (Elueze and Quan-Haase, 2018; Gallistl and Nimrod, 2020; Hänninen et al. 2021; Näsi et al. 2012; Mason and Pereira, 2011; Nimrod, 2017; Quan-Haase et al. 2018; Van-Deursen and Van-Dijk, 2011; Vulpe and Crăciun, 2020). Such differences have led to classifications that distinguish groups of older people according to whether or not they use the Internet (Van-Deursen and Helsper, 2015a), their digital knowledge, the ways they use online media (Quan-Haase et al. 2018; Vulpe and Crăciun, 2020), online search behaviour (Mason and Pereira, 2011), media consumption and entertainment preferences (Gallistl and Nimrod, 2020; Nimrod, 2017), and their concern for privacy in the digital environment (Elueze and Quan-Haase, 2018). Although these studies have made highly valuable contributions to a more precise definition of older internet users, they have not considered certain variables, nor specific uses made of ICT (e-government and e-commerce), which are decisive in defining silver surfer profiles. Moreover, such uses are vital in today’s developed societies in order for citizens to receive indirect benefits from internet use. In this regard, the study herein provides greater understanding of a segment of the population that is highly diverse in its digital behaviour, thereby complementing previous classifications of silver surfers that have differentiated profiles according to both their digital knowledge and their development of certain widespread uses of digital media (Quan-Haase et al. 2018; Vulpe and Crăciun, 2020). However, more specific and less extensive uses among seniors have been examined in this study, which are nevertheless essential nowadays in guaranteeing the full inclusion of seniors through e-commerce, e-government, online banking, and social networks. Moreover, in this study, trust and online security have been included as determining factors in making digital purchases and carrying out administrative procedures online.

The findings show that the majority of seniors have incorporated the Internet into their everyday lives, although significant differences still exist among the various groups. More than half of those surveyed claim to have an average level of digital skills (five clusters out of a total of seven). However, many still do not use the Internet fully nor efficiently, nor do they benefit from the enhanced autonomy and quality of life it can offer (Tirado-Morueta et al. 2016). The Able and Daring are the most numerous typologies, which reflects progress in the incorporation of seniors into the digital realm. However, neither of these clusters recognises having a high level of digital skill, as claimed by users in other classifications of silver surfers, including Savvy Users (Quan-Haase et al. 2018), Augmenters (Mason and Pereira, 2011) and Digitally Immersed Communicators (Vulpe and Crăciun, 2020). The Sceptical are in third place in terms of the number of individuals, and their use of ICT is largely limited to WhatsApp and e-mail. On the one hand, this silver surfer profile has certain similarities with the Reluctant (Quan-Haase et al. 2018), who have a low level of digital skills and limitations in their use of the Internet. On the other hand, the Sceptical also resembles the Fundamentalist Elueze and Quan-Haase, 2018), who has a lack of trust in carrying out any online task that involves sharing personal or bank data over the Internet.

With regard to socio-demographics, although Stockwell et al. (2020) define older adults who use technology as male, well-educated, and largely oriented toward maintaining contact with those already in their physical world, the findings of this paper reveal a profile that nearly achieves equality among silver surfers who make more diversified and specific use of the Internet (the Daring). Nevertheless, the idea that the Internet is male-dominated (Van-Deursen and Helsper, 2015a) has been reinforced, as the percentage of men in each group is higher than that of women, except for the Sceptical cluster, which is evenly divided between the genders. According to the results of this study, age is not a variable that implies large differences among groups of senior internet users, unlike the findings of Mason and Pereira (2011), which point to younger people as more digitally advanced. In general, the low impact of socio-demographic variables on the differentiation of profiles suggests that the uses made of the Internet by navigators and their digital skills are more important than other factors in achieving tangible results associated with online use (Van-Deursen and Helsper, 2018).

As a result, the ageing of developed societies requires a commitment from the various social, economic, public, and private actors to these vulnerable segments of the population (Jia et al. 2015; Kolotouchkina et al. 2022), along with a comprehensive approach that takes into account the users who are least capable and more insecure on the Web (the Insecure and Sceptical in this study). ICT and the Internet can raise awareness of ageing and the importance of seniors (Mosberg-Iversen and Wilin’Ska, 2018), and this could be accomplished by eliminating the negative stereotypes of old age (Makita et al. 2019). To be more specific, in countries that provide more opportunities for seniors to improve their skills, the perception of competence is connected to well-being (Fasel et al. 2020). As such, there is a need to work on developing the online expertise of silver surfers.

The generational digital divide is gradually decreasing, partly due to the current always on culture, but also because the digital world has become more familiar and attractive to older people (Lüders and Gjevjon, 2017). Generations born at the end of the first half of the twentieth century display a higher probability of using the Internet than those born earlier, due to having experienced natural contact with ICT (Gilleard and Higgs, 2008). Yet significant differences between internet users over the age of 60 persist, which highlights the continued existence of an internal digital divide, as noted in previous research (Elueze and Quan-Haase, 2018; Gallistl and Nimrod, 2020; Hänninen et al. 2021; Näsi et al. 2012; Mason and Pereira, 2011; Nimrod, 2017; Quan-Haase et al. 2018; Van-Deursen and Van-Dijk, 2011; Vulpe and Crăciun, 2020). Such findings have been reaffirmed by the results of the study herein. Technological literacy can promote more realistic digital inclusion (Abad-Alcalá et al. 2017) for less capable seniors (the Insecure and Sceptical), thereby reducing the digital divide among them (Lee and Porumbescu, 2019). Programmes for digital literacy should be designed with the aim of fostering a full, participative life for seniors (Abad-Alcalá, 2014). In this regard, proposals that involve more experienced seniors in the process of teaching-learning are of interest, as such programmes could make an important contribution to increasing the confidence of senior students in learning to use ICT (Woodward et al. 2013).

The findings of this study indicate progress with regard to the results of research in the early years of the 21st century, which showed that use of the Internet and ICT by seniors was much more basic than that of a true silver surfer (Selwyn et al. 2003). Nevertheless, there is still a long road to travel before the spread of technologically advanced profiles among older adults will be achieved, including Augmenters (Mason and Pereira, 2011), Heavy Asynchronous Media Users (Nimrod, 2017), Savvy Users (Quan-Haase et al. 2018), and Digitally Immersed Communicators (Vulpe and Crăciun, 2020). In spite of this, the use of technology can boost the confidence of seniors regarding their capabilities and empower them to make decisions in society (Sánchez-Valle et al. 2017). If seniors are equipped with tools that enable them to use the Internet and ICT, this will stimulate activity, which is associated with improved health (Näsi et al. 2012). Consequently, there is a pressing need to optimise and incentivise programmes of e-inclusion that will allow silver surfers to experience the full benefits of ICT and the Internet, especially due to the fact that nowadays performing purchases and administrative procedures online is a necessity. Those who do not have the knowledge to carry out these online tasks, or simply are unable to do so, face limitations that will affect their quality of life and restrict their rights. However, the findings of this article show a low level of trust among some older adults (the Sceptical and Insecure) toward online commerce and administration, as well as a perceived lack of security in the processing of personal data in the digital sphere, which prevents them from making greater use of online tasks that are increasingly essential. These a priori economic uses of the Internet end up being associated not only with monetary benefits for users, but also with tangible assets that are social, personal, and cultural, the achievement of which produces greater satisfaction (Van-Deursen and Helsper, 2018). Moreover, if the Internet is used in a constructive way, seniors might develop the four types of internet skills identified by Van-Deursen and Van-Dijk (2009, 2011), as older adults require basic skills that will allow them to carry out the following: Take advantage of various instrumental uses of the Internet (operational internet skills); navigate through the different pages and platforms (formal internet skills); attain competence in finding the information they need (informational internet skills); and develop strategic abilities for achieving objectives that will improve their standing in society (strategic internet skills).

With regard to the limitations of this research, the main constraint is the focus on one particular country, along with the specific variables related to its culture, economy and society, which may have influenced the definition of the profiles. This study provides a national perspective that enhances the results of macro studies such as that of Vulpe and Crăciun (2020), by incorporating nuances that enrich the explanation of senior behaviour in the digital realm. However, future research from a longitudinal and cross-cultural perspective is needed in order to explore the impact of contextual variables on technological use by older people. By extending the study of this topic to other countries, the profiles of silver surfers of different nationalities could be compared. In this way, similarities and differences could be obtained regarding the ways in which older internet navigators use ICT based on their place of residence and cultural variables. Therefore, future research is needed in order to complement the findings of this study and to refine the profiles of silver surfers, both at the local and international levels. As such, further progress could be made toward understanding the separate and main effect of country of origin on the preferences of older people in using the new media (Ivan et al. 2020; Loos and Ivan, 2022).

In addition, it must be acknowledged that the research presented in this article is based on a small sample. While the margin of error in this study is acceptable, it may be too high for the purpose of revealing reliable regional differences or ensuring the representativeness of less numerous profiles at the national level. Thus, further research based on the results of this study, but using a larger sample, would allow the profiles of silver surfers to be identified with more statistical precision.

Another shortcoming of this study is the fact that the cluster analysis does not prioritise variables of gender, social class, or educational levels, as the focus has been on the taxonomy of silver surfers according to the ways they use the Internet, and their behaviour on the Web as well. Consequently, future studies might consider investigating the classifications related to these types of variables. Furthermore, another restriction of this research is not having delved into the impact of design on the use of technology by seniors. In fact, other research has pointed out that design plays a key role in use by this social group. This is largely due to the difficulties experienced in navigating the Web, where design often complicates access and usability (Llorente-Barroso and Sáez-Díez-Rebanal, 2019). This situation could be resolved by bolstering interface designs adapted to the needs of seniors that take into account age-friendly criteria, which could add social and human value related to their needs (Yap et al. 2017). Among the design aspects that might foster the acceptance of online interaction by seniors, one that stands out is the structural simplification of websites (Llorente-Barroso and Sáez-Díez-Rebanal, 2019). In this regard, the TAM (Technology Acceptance Model) typifies a range of variables of perceived ease-of-use, and the usefulness of technology, which determine the attitude of seniors toward certain online tasks, such as e-government (Alzahrani et al. 2018; Chirara, 2018; Lee and Porumbescu, 2019; Sánchez-Valle et al. 2022), as well as e-commerce (Gopal and Murale, 2018; Smith, 2008; Viñarás-Abad et al. 2022). A change in website design to a more simplified structure (Abad-Alcalá et al. 2017) might also motivate the Insecure and Sceptical to make greater use of the Web.

Finally, it must be acknowledged that this research has not considered the consequences of the pandemic on the use of ICT by seniors. Some research has recognised the ramifications of the health crisis at the socio-emotional level, as ICT has brought older people closer to their families and friends, alleviating the negative effects of social isolation on their health (Llorente-Barroso et al. 2021). However, other studies have highlighted an increase in *technostress*, warning that ICT use could have a very different impact on seniors, depending on the profile of each user and the circumstances in which they find themselves (Nimrod, 2020).

## Supplementary information

SUPPLEMENTAL MATERIAL (DATA)

## Data Availability

All materials and data from this research are available upon request from the corresponding author.

## Footnotes

1 Euclidean, Chevyshev, Manhattan, Minkowski, Canberra, and Mahalanobis distances were attempted using the base and philentropy package (HG, 2018), as well as PCA, KODAMA, ISOMAP, and Shannon nonlinear mapping for dimensionality reduction. The best dissimilarity matrix was selected using the Hopkins statistic (clustertend package).

2 The KODAMA is a learning algorithm for unsupervised feature extraction and is specifically designed for analysing noisy and high-dimension datasets. KODAMA works in a way that is similar to algorithms such as t-SNE, Shannon Nonlinear Mapping, and ISOMAP, thereby reducing the dimensionality of the dataset in a non-linear way so that meaningful groups can be identified.

3 The silhouette width computes the distance between each point, and points in other clusters; connectivity measures the extent to which each observation and its neighbours are in the same cluster; the Dunn index is a ratio of the distance between each cluster and their size.

4 APN measures the average proportion of observations that are not placed in the same cluster using the full data and removing one column. AD measures the average distance between observations placed in the same situations. ADM measures the average distance between cluster centres for observations placed in the same cluster in both cases.
